# Safety and Efficacy of Compression Therapy to Prevent Chemotherapy-Induced Peripheral Neuropathy in Lower Extremities of Breast Cancer Patients: A Pilot Study

**DOI:** 10.7759/cureus.60998

**Published:** 2024-05-24

**Authors:** Mai Okazaki, Hiroko Bando, Azusa Terasaki, Aya Ueda, Akiko Iguchi-Manaka, Bryan J Mathis, Hisato Hara

**Affiliations:** 1 Department of Breast and Endocrine Surgery, University of Tsukuba Hospital, Tsukuba, JPN; 2 Department of Cardiology, International Medical Center, University of Tsukuba Hospital, Tsukuba, JPN

**Keywords:** chemotherapy-induced peripheral neuropathy, peripheral neuropathy, supportive care and symptom management, compression therapy, breast cancer

## Abstract

Background

Chemotherapy-induced peripheral neuropathy (CIPN) is a problematic adverse event for breast cancer patients receiving taxane antimitotic agents. We evaluated the effectiveness of compression therapy against CIPN in the lower extremities of breast cancer patients receiving taxanes.

Methods

Eligible patients scheduled for perioperative treatment with taxanes for early-stage breast cancer were enrolled. Each patient wore latex-free surgical gloves and compression socks, putting on two layers of each 15 minutes before the administration of taxanes and removing them 15 minutes after administration. Peripheral neuropathy (PN) was evaluated using the Common Terminology Criteria for Adverse Events (CTCAE) version 4.0 and the Patient Neurotoxicity Questionnaire (PNQ). The primary endpoint was the incidence of CTCAE version 4.0 grade 2 or higher CIPN in the lower extremities during the entire period of perioperative chemotherapy with taxanes.

Results

PN assessment by CTCAE in the lower extremities, the primary outcome, showed that 13.3% developed grade 2 sensory disturbances, and 8.3% developed grade 2 motor disturbances. The incidence of CTCAE grade 2 or higher PN in the hands was 26.7% for sensory disturbances and 13.3% for motor disturbances during the entire study period. No patient had grade 3 or higher PN. No adverse events due to compression therapy were observed.

Conclusion

Compression of the lower extremities with compression socks tended to reduce the incidence of CIPN compared to the general incidence. Compression therapy may help prevent the development of CIPN.

## Introduction

Chemotherapy-induced peripheral neuropathy (CIPN) occurs in 50% or more of breast cancer patients receiving taxanes. It involves axon degeneration, dysregulation of calcium homeostasis, ion channel disruption, and microtubule disruption in nerves, leading to symptoms such as burning pain, numbness, tingling, and impaired fine motor skills [1-3]. CIPN is devastating for patients due to both quality of life (QoL) degradation and the need for treatment interruptions that may reduce overall survival. It is most often seen during paclitaxel and docetaxel chemotherapy courses. Other drugs, such as platinum-based antineoplastics, vinca alkaloids, epothilones (ixabepilone), taxanes, proteasome inhibitors (bortezomib), and immunomodulatory drugs (thalidomide), also carry risks of developing CIPN [2,4]. CIPN is currently graded using the Eastern Cooperative Oncology Group (ECOG) criteria, focusing on the impact of CIPN on activities of daily living (ADL) since impairments in motor function can severely affect QoL [1].

According to the 2020 guidelines of the American Society of Clinical Oncology Clinical Practice (ASCO), drug therapy and physical therapies such as acupuncture, exercise, cold therapy, and compression therapy (ComT) for preventing the onset of CIPN are not recommended due to low evidence levels. Early diagnosis of CIPN and consideration of treatment changes are recommended [5]. The most frequent treatment strategy involves dose reduction or cessation of taxane-based drugs, which may affect the prognosis of breast cancer [3]. Although duloxetine is moderately recommended for treating CIPN, further investigation of effective treatment and prevention methods is still necessary [5,6].

Cold therapy works through localized vasoconstriction of the extremities, reducing drug exposure in the periphery and thereby lowering the risks of CIPN. ComT, similarly, reduces peripheral blood flow through pressurization and subsequent vasoconstriction. Several studies using surgical gloves to compress the hands of breast cancer patients who received nanoparticle albumin-bound paclitaxel (nab PTX) reported protection against CIPN comparable to that of cold therapy [7-9]. However, the utility of ComT suffers from conflicting evidence, and there is a total lack of evidence regarding the effect of foot compression on CIPN.

Here, we present an interventional clinical trial of ComT on the hands and lower extremities of breast cancer patients with CIPN receiving taxanes over a three-month period. We expected that all patients would complete the therapy sessions, and information on the glove/sock types, sizes, and pressure variables will be of significant benefit for developing treatment guidelines.

## Materials and methods

Study design

This trial was conducted at the University of Tsukuba Hospital in Japan from October 2021 to September 2022. It was a single-center, prospective, interventional, single-arm, open-label study. This study was registered in the Japan Registry of Clinical Trials under the identifier jRCTs 032210221 and was conducted in accordance with the Declaration of Helsinki. All patients provided written informed consent.

Patients

Women with pathologically diagnosed primary breast cancer receiving neoadjuvant or adjuvant chemotherapy were eligible for this study. Inclusion criteria were age 20-70 years and an ECOG performance status of 0-1. Patients were excluded if they had peripheral neuropathy (PN) in the hands and/or feet, arterial blood circulation disorders (such as arteriosclerosis obliterans), infections or inflammatory diseases on the skin around the hands and/or legs, severe congestive heart failure, or if diagnosed with acute deep vein thrombosis or thrombophlebitis of the legs.

Procedure

This study used surgical gloves (Sensi Touch Pro or Sensoprene Green, Toray Medical, Japan) and compression socks (Jobst so Soft 30, Jobst, Germany). The optimal compression glove size was one surgical glove size smaller than the size determined by the patient. Since the method of lower extremity compression therapy for preventing CIPN has not been established, medical elastic stockings were used as a safety measure. The optimal size for compression socks was determined by the circumference of the patient's ankles and calves. After sizing, a manometer (Picopress, MICROLAB, Italy) was used to measure pressure on the back of the hand and 5 cm above the ankle. The lower extremity compression pressure was set to high pressure (34-46 mmHg), recommended for treating lower extremity lymphedema [10].

Each patient wore latex-free surgical gloves and compression socks, applying two layers of each 15 minutes before the administration of taxanes and removing them 15 minutes after. Patients wore these compressive devices for up to 90 minutes at a time. To avoid peroneal nerve palsy, no pressure was applied around the fibular head when wearing compression socks.

Patients received four cycles of weekly paclitaxel at 80 mg/m2 or four cycles of docetaxel at 75 mg/m2 every three weeks. Combinations of anti-HER2 therapy and carboplatin (AUC6) every three weeks were allowed. Patients receiving carboplatin underwent six cycles of docetaxel at 75 mg/m2. Four patients were treated with anthracycline-cyclophosphamide combination therapy before taxanes. Medication to relieve PN symptoms was allowed.

PN was evaluated using the Common Terminology Criteria for Adverse Events (CTCAE) version 4.0 and the Patient Neurotoxicity Questionnaire (PNQ) at the 1st, 4th, 7th, and 10th doses of paclitaxel or each dose of docetaxel, at the end of chemotherapy, and three months later. The PNQ is a tool developed for patients to self-assess the onset and severity of CIPN and includes ratings for sensory and motor disturbances [4,11]. Patients were evaluated for each disorder on a 5-point scale from A (no neuropathy) to E (severe neuropathy). Grades A to C indicate no significant effects on activities of daily living (ADL), but D and E indicate significant problems with ADL. In our study, we converted the A to E grades into numeric scores ranging from 1 to 5.

Outcomes

The primary endpoint was the incidence of CTCAE version 4.0 grade 2 or higher CIPN in the foot after perioperative chemotherapy with taxanes. The secondary endpoints included the incidence of PN in the hands after completion of perioperative chemotherapy with taxanes, evaluation of CIPN in the hands and feet using the PNQ, completion rate of foot compression therapy, period until CIPN in the hands and feet reached grade 2 or higher, compressive pressure measurements in the hand and foot, and the incidence of skin disorders in the hands and feet. At the end of the study, patients also completed the Patient Questionnaire Survey on Compression Therapy, which gathered information on the difficulty of wearing the device, discomfort and pain from compression therapy (rated on a scale of 0 to 10), and their overall impressions.

Statistical analysis

Assuming a control group, the incidence of CIPN in the control group was expected to be similar to that reported in previous studies. Therefore, in this study, we did not establish a control group. Endpoints were analyzed descriptively and expressed as medians or means for continuous variables. Due to small sample sizes, we conducted exploratory analyses on the incidence of compressive pressure and skin disorders.

## Results

Patient demographics

A total of 10 patients were enrolled in the study, with a median age of 50 years (range: 32-63 years) at the start of chemotherapy. All patients were ECOG status 0 at the beginning of the study. Two patients received four cycles of weekly paclitaxel, while eight patients received docetaxel. Of those who received docetaxel, four underwent four cycles of docetaxel alone, and four received docetaxel in combination with trastuzumab and pertuzumab. Additionally, two received six cycles of docetaxel, trastuzumab, pertuzumab, and carboplatin. All patients completed scheduled chemotherapy, but one patient received a reduced dose of docetaxel due to malaise and poor physical condition associated with chemotherapy. Glove sizes used were size 5.5 for seven patients and size 6.0 for three patients, with sock sizes of S for one patient and M for nine patients. One patient changed from size S to M because the sock felt too tight. Patient characteristics and compression device information are summarized in Table 1.

**Table 1:** Patient characteristics. ECOG: Eastern Cooperative Oncology Group.

Patient, n	10
Median Age, y (range)	50 (32-63)
BMI (range)	21.7 (17.8-27.7)
ECG Performance Status	
0	10
1	0
Treatment, n	
Neo-Adjuvant	7
Adjuvant	3
Primary Tumor Subtypes, n	
Luminal	4
Luminal HER2	3
HER2	1
Triple-Negative	2
Taxane Type, n	
Paclitaxel	2
Docetaxel	8
Combination Therapy with Trastuzumab and Pertuzumab	4
Combination Therapy with Calboplatin	2
Average Cumulative Taxane Dose, mg/m^2^	
Paclitaxel	960
Docetaxel	337.5
Size of SG, n	
5.5	7
6.0	3
Size of Compression Socks, n	
S	1
M	9

CIPN incidence

**Figure 1:**
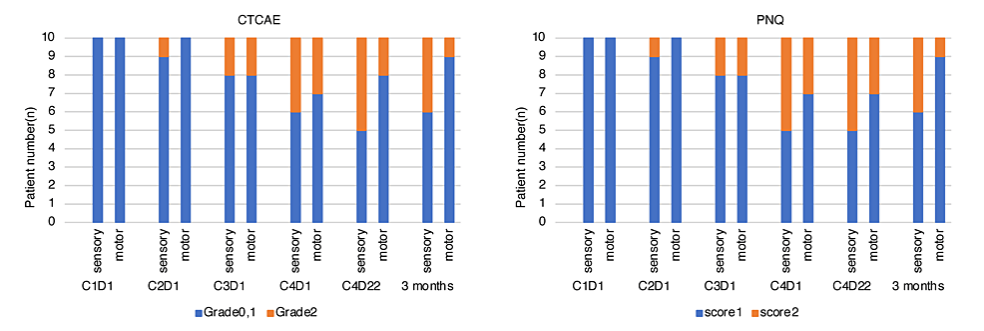
CIPN in the hands by grade: sensory and motor. CTCAE: Common Terminology Criteria for Adverse Events; PNQ: Patient Neurotoxicity Questionnaire; C1D1: Day 1 of 1st Cycle of Chemotherapy, Cycle 1 Day 1; CIPN: Chemotherapy-induced peripheral neuropathy.

CTCAE and PNQ sensory and motor grades for hand PN are shown in Figure 1. The incidence of CTCAE grade 2 or higher PN in the hand was 26.7% for sensory and 13.3% for motor disturbances during the entire study period. No patient had grade 3 or higher PN. Three months after the end of chemotherapy, four patients had grade 2 sensory disturbances, and one patient had a residual motor disturbance. PN assessment by CTCAE of the lower extremity, the primary outcome, showed that 13.3% developed grade 2 sensory disturbances, and 8.3% developed grade 2 motor disturbances. Three months after the end of chemotherapy, one patient still had a grade 2 sensory disturbance, but no patient had grade 2 or higher motor disturbances.

**Figure 2:**
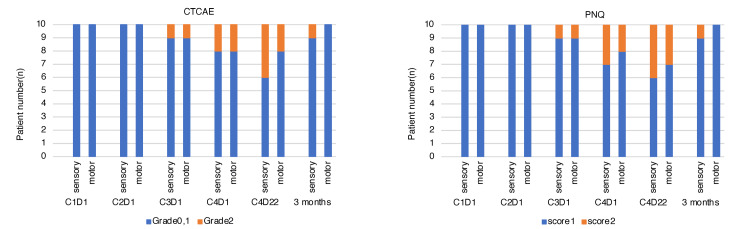
CIPN in the lower extremities by grade: sensory and motor. CTCAE: Common Terminology Criteria for Adverse Events; PNQ: Patient Neurotoxicity Questionnaire; C1D1: Day 1 of 1st Cycle of Chemotherapy, Cycle 1 Day 1; CIPN: Chemotherapy-induced peripheral neuropathy.

The incidence of PN as assessed by PNQ was 28.3% for hand sensory disturbances with a score of ≥2, and 15.0% for motor disturbances. For the lower extremities, sensory disturbances were 15.0% and motor disturbances were 10.0%, which tended to be higher than those evaluated by CTCAE. No patient had a PNQ score of 3 or higher. Among those with a PNQ score of ≥2, the median time to onset of PN symptoms was 9 weeks for both sensory and motor disturbances in the hands and feet. These results are summarized in Figure 2.

All patients completed hand and foot compression therapy. None of the patients experienced significant changes in calf and ankle circumference during the entire study, nor did they experience discomfort from compression therapy. Two patients received medication for CIPN: one patient received oral pregabalin, and another received goshajinkigan in efforts to prevent CIPN. None of the patients continued these medicines after the three-month study period.

Pressure results

**Figure 3:**
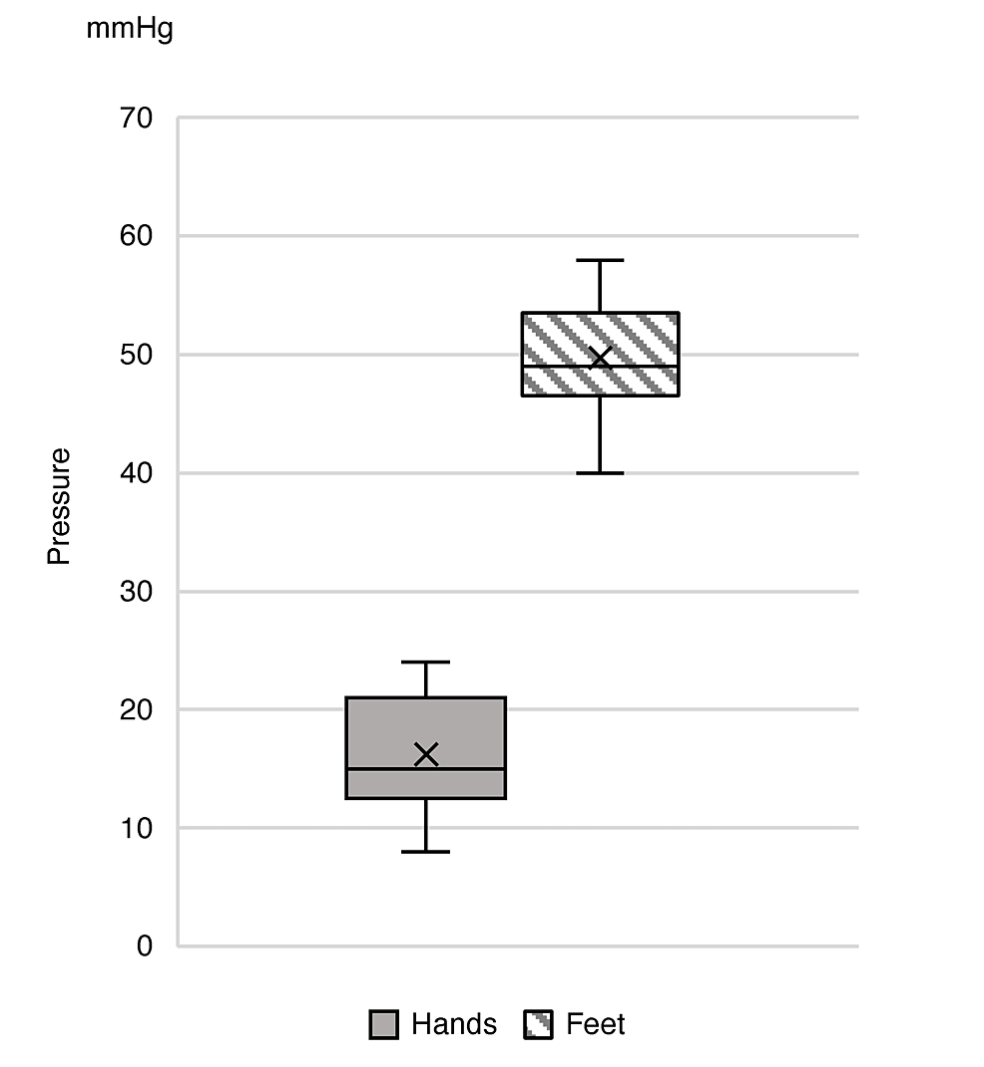
Compression pressure on the hands and lower extremities.

A manometer was used for compressive pressure measurement in the hand and foot with SG and CS. The median pressure exerted by the SG on the back of the hand was 15 mmHg (range: 8-24 mmHg), and the median pressure 5 cm above the ankle with the CS was 49 mmHg (range: 40-58 mmHg). No patient changed glove or sock size after study initiation, so compression pressure was not remeasured. Results are summarized in Figure 3.

Skin disorders/Adverse events

We investigated the incidence of skin disorders suspected as adverse effects of taxanes. A total of three patients had exfoliation of the hand, and one had exfoliation of both the hand and foot. All cases were grade 1 and did not require treatment. No adverse effects on the skin due to wearing compression devices were observed.

Discomfort scores and patient quotes

Questionnaire responses were obtained from all patients participating in the trial. Only two patients required help to remove gloves and socks. Compression therapy had a mean discomfort score of 1.8 in the hand and 2.2 in the foot, and no patient complained of pain from compression therapy. Representative impressions obtained from the patients were generally positive, mentioning only temperature as a discomfort. Some quotes from patient responses (translated into English) are: "Thanks to compression therapy, my peripheral neuropathy symptoms were mild." "It was difficult to put on or take off the second pair of gloves and socks." "It was hot due to wearing surgical gloves and compression socks."

## Discussion

Our study contributes to the limited evidence supporting the utility of ComT for CIPN by confirming its safety profile and reporting no advanced grades of CIPN, indicative of the protection afforded by compression. Furthermore, no adverse events such as pain or skin disorders related to compression therapy were observed, and the therapy was simple to implement. Over the study period and a three-month follow-up, all patients were able to complete the study, and subjective impressions were generally favorable. CIPN is a potentially debilitating side effect of taxane-based chemotherapy, and compression therapy may be a useful, albeit relatively unknown, alternative to cold therapy [12].

Although the effectiveness of cold therapy has been reported in diverse studies, patient discomfort could impact compliance [13]. Compression therapy, on the other hand, operates on a similar principle of vasoconstriction to reduce exposure of nerves to taxane-based chemotherapy but carries less discomfort. However, the European Society of Medical Oncology (ESMO) 2020 guidelines are similar to those of ASCO, with a recommendation grade of C and an evidence level of III for prophylactic physical therapy for CIPN, indicating that the evidence for recommendation is considered insufficient. Therefore, there is a need to accumulate evidence regarding the effectiveness of ComT in preventing CIPN while also increasing awareness to promote more clinical studies that test both ComT and combination cold and compression therapies for moderate-to-severe CIPN [12].

In the Kamigata study conducted by Tsuyuki S et al. et al., compression therapy using surgical gloves on the peripheral hand significantly lowered the incidence of sensory disturbances of CTCAE grade 2 or higher (21.4% vs. 76.1%) and motor disturbances (26.2% vs. 57.1%). In this study, none of the patients presented with CIPN grade 3 or higher PN symptoms, and CTCAE grade 2 or higher sensory (hands: 26.7%, feet: 13.3%) and motor impairments (hands: 13.3%, feet: 8.3%) tended to occur less often than the general frequency [7]. Current ComT evidence carries some controversy, as several studies, including one by Kotani H et al., have reported negative results regarding CIPN. Notably, Kotani H et al. reported no significant effect of ComT with surgical gloves on paclitaxel-induced peripheral neuropathy; however, results were reported after excluding patients who developed grade 2 neuropathy or higher from the study [14]. That study did not follow up with patients and was restricted to one taxane-based drug. Our results, in contrast, demonstrated at least some efficacy in preventing higher grades of CIPN, and a three-month follow-up verified the efficacy of ComT with socks and gloves against CIPN after treatment.

Methodology is an important factor in ComT for reproducibility, particularly as there are currently no established guidelines for CIPN. It is known that upper extremity ComT to prevent CIPN can be performed by wearing two surgical gloves, but no prospective studies have simultaneously explored the degree of compression (measured in mmHg) and the effects of lower extremity compression therapy. Therefore, to perform lower extremity compression therapy safely, the degree of compression was initially estimated based on reports from lymphedema trials. Lymphedema guidelines classify compression pressure into three stages, low, medium, and high pressure, for both upper and lower extremities. Since there were no analogous studies for our protocol development, we assumed a high pressure (34-46 mmHg) to be a feasible pressure that could sufficiently restrict blood flow. Additionally, large individual differences in finger length, thickness, and hand size were factors in choosing glove sizes for testing. Although peroneal nerve palsy was expected as an adverse event of lower extremity compression therapy, none of the patients experienced this condition either during the study or at follow-up. This was anticipated, as peroneal nerve palsy is caused by prolonged pressure near the fibular head, and its frequency is as low as 0.3% [15]. For the feet, treatment duration was a safety consideration, as compression by elastic stockings is considered a risk factor for compartment syndrome, reported to increase during long-term surgeries exceeding three hours [15, 16]. In our study, the risk of developing peroneal nerve palsy was considered low as the patient was able to move the lower extremities freely and wearing time was restricted to 90 minutes. Although our method may have increased the risk of insufficient hand compression in this study, future explorations into the relationship between compression pressure, the incidence of CIPN, and the optimal pressure index will help standardize values for ComT in both upper and lower limbs.

This study must acknowledge limitations in the form of its single-arm, single-center design and a low number of patients. Additionally, patients were not grouped by doses of taxanes, and our design relied on commercially available equipment (gloves) not intended for compression use. Objective evaluations, such as electrodiagnostic testing, which were not conducted for this study, may also be useful in measuring the true effect of ComT [17]. Therefore, future multicenter studies that address these limitations and demonstrate the utility of ComT are highly desired.

## Conclusions

In a population of Japanese, female, middle-aged breast cancer patients receiving taxanes, CIPN symptoms were grade 2 or less with the combination of ComT. There were no adverse events and compression pain was well controlled. This study adds to the body of evidence that ComT can safely help prevent CIPN symptoms.
